# Combining stable isotopes, morphological, and molecular analyses to reconstruct the diet of free‐ranging consumers

**DOI:** 10.1002/ece3.6397

**Published:** 2020-05-27

**Authors:** Michaël Bonin, Christian Dussault, Joëlle Taillon, Nicolas Lecomte, Steeve D. Côté

**Affiliations:** ^1^ Caribou Ungava Centre d’études nordiques Université Laval Québec QC Canada; ^2^ Direction de l’expertise sur la faune terrestre, l’herpétofaune et l’avifaune Ministère des Forêts, de la Faune et des Parcs Québec QC Canada; ^3^ Chaire de recherche du Canada en écologie polaire et boréale Université de Moncton Moncton NB Canada

**Keywords:** *Canis lupus*, carnivores, diet reconstruction, feeding strategies, molecular diet analyses, morphological diet analyses, omnivores, stable isotopes, trophic interactions, *Ursus americanus*

## Abstract

1. Accurate estimates of animal diet composition are essential to untangle complex interactions in food webs. Biomarkers and molecular tools are increasingly used to estimate diet, sometimes alongside traditional dietary tracing methods. Yet only a few empirical studies have compared the outcomes and potential gains of using a combination of these methods, especially using free‐ranging animals with distinct foraging preferences.
2. We used stable isotopes, morphological, and molecular analyses to investigate the diet of free‐ranging consumers with two distinct diet types, that is, carnivore and omnivore. By combining the three analytical methods to assess the diet of consumers during the same period, we aimed to identify the limits of each method and to assess the potential benefits of their combined use to derive diet estimates.
3. Our results showed that the different methods led to a consistent diet description for carnivores, which have a relatively simple diet mixture, but their outcomes somewhat differed for omnivore, which have a more complex diet. Still, the combined use of morphological and molecular analyses enhanced the diversity of food sources detected compared to the use of a single method independently of diet types. Precision of diet estimates derived from stable isotope analyses was improved by the addition of priors obtained from morphological and molecular diet analyses of the same population.
4. Although we used free‐ranging animals without a known diet, our empirical testing of three of the most widely used methods of diet determination highlights the limits of relying over a single approach, especially in systems with few or no a priori information about the foraging habits of consumers. The choice of an appropriate approach of diet description should be a key step when planning dietary studies of free‐ranging populations. We recommend using more than one dietary determination methods especially for species with complex diet mixtures.

## Graphical abstract

Estimates of diet composition are essential to decipher complex interactions in food webs. Combining approaches of diet reconstruction gains in popularity as it could bring complementary information about consumers' food habits. Still, there are few empirical examples of the outcomes of different methods applied to a consumer diet over the same period. Here, we compared the use of stable isotopes, morphological, and molecular analyses over a same period to reconstruct the diet of free‐ranging consumers with distinct diet compositions, that is, carnivore and omnivore. Although we found good agreement among methods for ranking the main food sources, their outcomes in terms of diet diversity diverged especially for rare food sources, which explain why divergence between methods was higher for the species with more complex diet mixture. In line with other studies, we argue that the choice of approaches for diet reconstruction should be carefully questioned in regards of research questions, time scales, as well as food habits of targeted consumers' species. Combining approaches should be regarded as an option ensuring the optimal coverage of diet composition when studying free‐ranging populations, especially when dealing with systems with few a priori knowledge about potential food sources and diet composition of consumers.

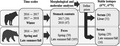

## 1 INTRODUCTION

Accurate estimates of diet composition are essential to untangle complex interactions in food webs, as well as to decipher the responses of species to global changes, but are hard to acquire especially in free‐ranging conditions (Araujo, Bolnick, & Layman, 2011; Bolnick, Yang, Fordyce, Davis, & Svanbäck, 2002; Nielsen et al., 2018). The variety of dietary tracing methods available, including morphological examination of remains found in feces and stomach contents, DNA barcoding, and biomarkers such as stable isotopes and fatty acid ratios, complexifies the choice of the most appropriate method in regards of specific research or management questions. The idea of using joint approaches has recently gained in popularity, especially to overcome the respective limitations of each dietary tracing method used alone (Horswill et al., 2018; Matley et al., 2018; Nielsen et al., 2018).

A recent review by Nielsen et al.(2018) on dietary tracing concluded that empirical studies investigating the respective limits and strengths of combined approaches to define food choices of consumers are lacking. Most studies, especially those of free‐ranging populations, rely on a single method of diet reconstruction making it difficult to show the gains linked to a joint use of multiple methods (but see Horswill et al., 2018; Jeanniard‐du‐Dot, Thomas, Cherel, Trites, & Guinet, 2017; O'Donovan, Budge, Hobson, Kelly, & Derocher, 2018; Tverin et al., 2019).During the last decades, diet estimates have mainly been derived from visual examination of undigested remains in feces and/or stomach contents (Steenweg, Gillingham, Parker, & Heard, 2015). The popularity of this approach mostly relies on its quick application and the possibility to obtain both quantitative and qualitative information on the diet, as well as assessing characteristics of food sources such as prey age, size, or development state (Klare, Kamler, & Macdonald, 2011). Diet estimates obtained with this approach can be expressed through either quantitative (per cent volume or mass of remains and relative biomass ingested) or qualitative (frequency or per cent of occurrence) metrics (Klare et al., 2011). While quantitative metrics would lead to more accurate estimates of the true consumers' diet, qualitative ones are more widely used for making comparisons among studies and methods. Qualitative estimates such as frequency of occurrence are also useful to document the range of potential food sources for a given species (Klare et al., 2011). Independently of the metrics used, dietary estimates derived from morphological analyses could be biased by differential digestibility of food items and low occurrence of certain food sources resulting in false negatives or unidentifiable items (Morin et al., 2019; Steenweg et al., 2015). For instance, although behavioral observations often reveal predation by rodents on the eggs of seabirds, it is difficult to accurately assess the importance and occurrence of this food source because eggshells and yolk rarely leave remains in feces of rodents (Drever, Blight, Hobson, & Bertram, 2000). Estimates based on morphological analyses could also be limited in their taxonomic resolution, especially in systems with closely related food sources leaving fewer traits to distinguish among species.

Molecular tools have emerged as an appealing solution to inform dietary analyses with high taxonomic resolution of food items without the need of relying on visually identifiable remains (De Barba et al., 2014; Pompanon et al., 2012; Quasim, MacDonald, & Sarre, 2018). In particular, DNA barcoding, which provide diet estimates based on the sequencing of DNA available in stomach contents and feces of consumers, has been used successfully in a variety of taxa (Carreon‐Martinez, Johnson, Ludsin, & Heath, 2011; Clare, Fraser, Braid, Fenton, & Hebert, 2009; Egeter, Bishop, & Robertson, 2015; Méheust, Alfonsi, Le Ménec, Hassani, & Jung, 2014; Waraniak, Baker, & Scribner, 2018). This approach has revealed higher or similar detection rates compared to the traditional analyses of undigested remains while also surpassing morphological analyses for its taxonomic resolution of detected food sources (Gosselin, Lonsinger, & Waits, 2017; Jeanniard‐du‐Dot et al., 2017). Still, controlled studies have shown that the main current limitation of molecular tools in dietary studies is the poor relationship between read counts (i.e., the number of DNA fragments detected per food taxa) and the food source biomass in the diet (Deagle et al., 2019; Lamb et al., 2019). Yet relative abundance of read counts could only be used in controlled conditions or when the number of potential food taxa is expected to be small (Deagle et al., 2019). As of now, diet estimates from molecular analyses have rarely been used in a quantitative way, and reporting the frequency of occurrence of food taxa (qualitative diet description) is often selected as a conservative option (Alberdi et al., 2019; Lamb et al., 2019). Sample quality (usually measured as the DNA degradation rate due to environmental conditions), heterogeneous distribution of DNA in samples, and access to a reliable DNA reference database are additional challenges linked to the use of molecular tools to reconstruct the diet of consumers (Alberdi et al., 2019; Mata et al., 2019; Mumma et al., 2016). Considering the respective limitations of morphological and molecular analyses, studies of species with unknown diet would take advantage of a combined approach, thereby increasing the coverage of potential diet items (Méheust et al., 2014; Xavier et al., 2018). Up to now, many studies comparing the two approaches have been conducted using known diets (controlled conditions) or over a subset of potential food sources for wildlife (Egeter et al., 2015; Granquist, Esparza‐Salas, Hauksson, Karlsson, & Angerbjorn, 2018; Mumma et al., 2016; Shores, Mondol, & Wasser, 2015). Hence, comparing respective outcomes for those methods in free‐ranging populations would bring additional cues to help identify the best method or combination of methods to use in order to address specific questions regarding wildlife diet.

As a robust analytical method to study diet, stable isotopes have gained a central role in ecology (Carreon‐Martinez & Heath, 2010; Dalerum & Angerbjorn, 2005; Hopkins, Kurle, & Davey, 2016). Compared to shorter time windows derived from the analysis of feces and stomach contents, one of the main benefits of stable isotope analyses (SIA) is that they provide quantitative dietary information over a broad range of time scales, as consumer tissue‐specific growth rates reflect individual foraging history from previous days (serum and liver) to months (hair and feathers) (Dalerum & Angerbjorn, 2005). It also allows either to track temporal changes in foraging behavior or to detect foraging tactics difficult to distinguish using feces or stomach contents unless large sample sizes are achieved (Edwards, Derocher, Hobson, Branigan, & Nagy, 2011; Watts & Newsome, 2017). On the other hand, SIA hardly reach the taxonomic resolution achievable with morphological and molecular methods, as isotopic ratios of similar food sources often overlap (e.g., broad food categories such as plants), making the distinction of their respective contribution to the diet of consumers a challenging task (Caut, Angulo, & Courchamp, 2008; Codron et al., 2012; Parnell, Inger, Bearhop, & Jackson, 2010). The optimal use of SIA also requires a minimum of a priori information on the consumer diet and/or on potential food sources available, questioning its use as a single approach to describe diet (Caut et al., 2008; Phillips et al., 2014). Such a priori information, used as priors in Bayesian‐based SIA (Phillips et al., 2014), can be derived from the literature or preferably from results obtained with other methods of diet description in the same consumer populations, thereby allowing considering regional specificities in food habits (Chiaradia, Forero, McInnes, & Ramirez, 2014; Franco‐Trecu et al., 2013; Swan et al., 2020). Because many studies are relying solely on SIA to determine the dietary choice of targeted species (but see, e.g., Horswill et al., 2018; O'Donovan et al., 2018), it is highly relevant to investigate how diet estimates based on SIA match those derived from morphological and molecular approaches for a shared period of time.

Here, we evaluated the agreement and complementarity of morphological identification of undigested remains, DNA barcoding, and stable isotopes over the same time scale to determine the diet of free‐ranging gray wolves (*Canis lupus*) and black bears (*Ursus americanus*) in northern Québec and Labrador, Canada. Diet of both species has been extensively studied along various landscapes, and both species are of wide management concerns (Kirby, Alldredge, & Pauli, 2016; Merkle, Polfus, Derbridge, & Heinemeyer, 2017; Newsome et al., 2016; Petersen & Ciucci, 2003; Welfelt, Beausoleil, & Wielgus, 2019; Zager & Beecham, 2006). We examined whether dietary information derived from multiple approaches would provide a more thorough description of consumers' food habits compared to the use of a single approach. When comparing morphological and molecular analyses, we hypothesized that molecular analyses would enhance the detectability of rare food sources and predicted that it would result in a higher diversity and taxonomic resolution of food sources. However, morphological analyses should provide some insights about the characteristics of food sources (e.g., age) that are impossible to obtain with molecular analyses. Because wolves have a relatively simpler diet mixture compared to black bears, we expected higher agreement in terms of diversity and ranking of food sources for them (a carnivorous versus. omnivorous diet). Finally, we expected that including priors derived from the same consumer populations over the same time scale would improve the precision of diet estimates determined through SIA as it would allow to consider regional specificities in food habits of consumers.

## 2 MATERIALS AND METHODS

### 2.1 Study design and sampling periods

**FIGURE 1:**
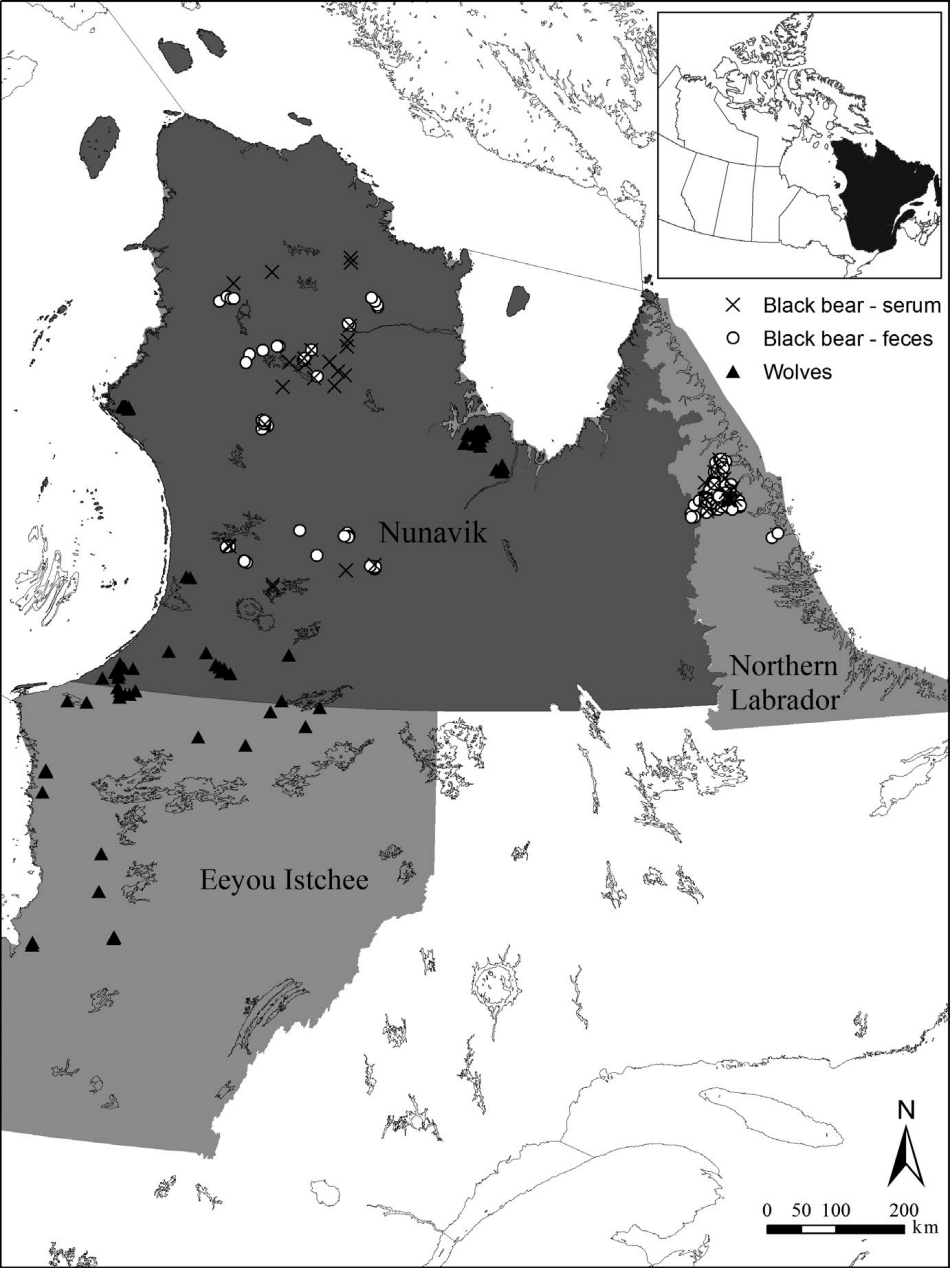
Study area in northern Québec and Labrador (Canada) showing the sampling locations for wolves and black bears. Within northern Québec, two subregions, Eeyou Istchee and Nunavik, are delineated based on administrative boundaries. See Figure 2 for sample sizes

**FIGURE 2:**
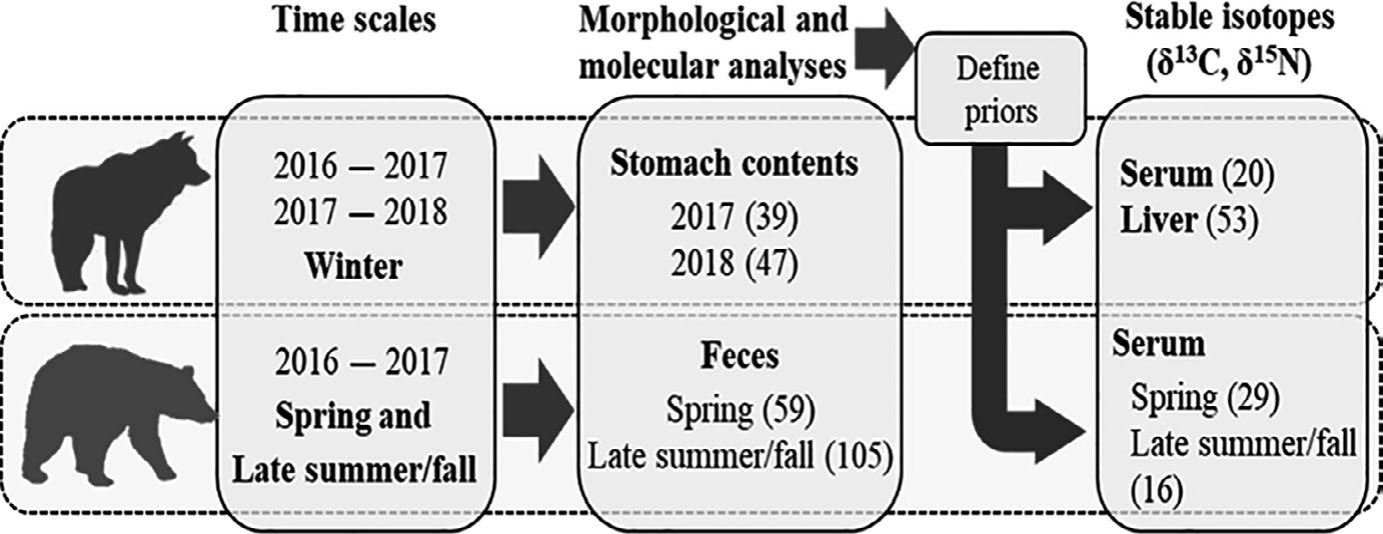
Summary diagram showing sample sizes available for the combined use of morphological, molecular, and stable isotope analyses to determine the diet of wolves and black bears in northern Québec and Labrador, Canada. Numbers in parentheses refer to sample sizes for each species–tissue combination

This study was part of a larger research project on the ecology of wolves and black bears in northern Québec and Labrador, Canada (Figure 1). Captures and handling of wildlife complied with the rules of the Canadian Council on Animal Care, and procedures were approved by Laval University and the Québec Ministère des Forêts, de la Faune et des Parcs Animal Care Committees (CPA‐FAUNE 16‐01, 17‐04, 18‐24, 19‐06, 19‐17). We applied three of the most common methods of diet reconstruction, that is, morphological examination of stomach and feces contents, DNA barcoding (hereafter referred as molecular analyses), and stable isotopes to determine the diet of both species at the population scale (Figure 2).

### 2.2 Wolves and black bears

We sampled stomach contents, liver tissue, and serum for wolves during winters (December‐April) of 2016–2017 and 2017–2018 (Figure 2). We collected black bear samples during spring (mid‐June) and late summer/fall (late‐August/mid‐September) in 2016 and 2017 (Figure 2). During each season of both years, we also sampled feces of black bears (Figure 2) at cluster sites delineated from the locations of bears fitted with GPS collars (11 bears in 2016 and 20 in 2017). We visited the clusters within 14 days of their use by a bear. Part of the samples (60%) for wolves were obtained through the traditional harvest of Cree and Inuit communities of northern Québec.

### 2.3 Food sources

We collected potential food sources for both species during different periods of the year. The main potential food sources available to wolves during winter were migratory caribou (*Rangifer tarandus*), muskoxen (*Ovibos moschatus*), moose (*Alces americanus*), beaver (*Castor canadensis*), ptarmigan (*Lagopus* sp.), and small‐ to medium‐size mammal species, for example, lemmings (*Lemmus* spp.) and hares (*Lepus* sp.). In addition, we seasonally collected a variety of animal and plant sources to encompass the range of food items potentially available to bears in each season: birds and bird eggs, fishes, grasses, forbs, herbaceous plants, and berries. We collected hair and/or muscle tissue samples for animals and complete aerial parts for vegetal food sources (See Appendix 1 for additional details on food sources).

### 2.4 Identification of undigested remains and DNA barcoding

We identified undigested remains in stomach contents of wolves and feces of black bears at the species level for mammals and at the order level for birds. Additionally, we distinguished calves from adult migratory caribou based on medulla patterns of hair found in feces of black bears collected in spring. For black bears, we randomly selected 10 samples of feces for which we identified food sources using the point‐frame method (100 intersection points, Ciucci, Tosoni, & Boitani, 2004), and a traditional examination method separating all items manually and doing visual estimation of proportions on a 100 cells check plate on which the sample is spread (hereafter called visual proportions). Because the proportions of food sources estimated using both methods were highly correlated (e.g., *r* = 0.8 for berries, *r* = 0.9 for small mammals), we decided to rely only on the point‐frame method to determine the diet of black bears given the significant reduction of time spent per sample (*t*_1,10_ = 7.75, *p* < .001) using the point‐frame approach (48 ± 18 min, mean ± *SD*) versus visual proportions (153 ± 60 min). We express diet estimates based on morphological analyses as frequency of occurrence (%FO) of each food source, that is, the number of samples where the food source *i*occurred divided by the total number of samples analyzed. We also calculated proportions of dry matter ingested (%P_DM_) for black bears based on intersection counts and corrected using digestibility correction factors from Baldwin and Bender (2009). The same observer counter‐validated all identifications.

We sent duplicate samples of feces (black bear) and triplicates of stomach contents (wolf) to the Canadian Centre for DNA Barcoding, Ontario, Canada, for detection of invertebrates, vertebrates, and plants based on DNA fragments. Each sample consisted of a homogenate of three subsamples taken in different locations from the original sample (before morphological analyses) to consider potential heterogeneity in prey DNA distribution (Alberdi et al., 2019; Mumma et al., 2016). Amplification steps were performed in duplicates using both concentrate and diluted DNA from the whole homogenate and were visualized by gel electrophoresis. Each sample was analyzed for vertebrates and plants DNA using specific primers targeting a 185‐base pair (hereafter bp) fragment of the *COI*region for vertebrates and a 163 bp fragment of the chloroplast *rbcLa* barcode region for plants. Each sample was tagged with IonXpress Universal Molecular Identifiers (UMIs). Sequencing was performed using an Ion Torrent S5 high‐throughput sequencer. The resulting sequence reads were associated with their source sample using UMIs, filtered to remove low quality reads, trimmed to remove primer, and then filtered again for a minimum size of 100 bp. Assignation of the filtered reads was carried using the BOLD reference library and the BLAST algorithm (Ratnasingham & Hebert, 2007). Identification was only accepted as genuine if they were supported by a minimum of 50 reads that matched a reference sequence with a threshold of 95% identified across at least 100 bp. Due to the lack of species‐specific reference barcode regions for rare or elusive species in arctic habitats, we limited our assignations to family level and regrouped food sources upon coarser categories such as small mammals and plants and berries to allow comparison with occurrence data from morphological analyses. Still, we retained finer taxonomic resolution for qualitative diet estimates using the molecular analyses. We tallied occurrence data across replicates for each food source and reported the results of molecular analyses as %FO. Finally, we combined the results of morphological and molecular analyses by tallying the occurrence of food sources detected by either technique for each original sample which were processed through both types of analyses. We considered these combined diet estimates as the most representative picture of the detectable diet diversity achievable for wolves and bears in our study area. We used these estimates to guide the selection of food sources for SIA.

### 2.5 Stable isotopes analyses

We used the isotopic signature of serum (wolves and bears) and liver (wolves only) samples to assess the diet of both species over the same time scale covered by morphological and molecular analyses (Figure 2). Samples of consumers and food sources were simultaneously analyzed for nitrogen (δ^15^N) and carbon (δ^13^C) isotopic ratios at the Laboratoire d’Océanographie of Laval University, Québec, Canada. Isotopic analyses were performed by continuous‐flow isotope ratio mass spectrometer (Thermo Electron Delta Advantage) in the continuous‐flow mode (Thermo Electron ConFlo III) using an ECS 4010 Elemental Analyzer/ZeroBlank Autosampler (Costech Analytical Technologies). Measurement precision was ±0.2‰ for δ^13^C and ±0.1‰ for δ^15^N.

To account for trophic discrimination in SIA, we used trophic discrimination factors (TDFs) of 4.5 ± 0.3‰ (δ^15^N) and 2.2 ± 0.3‰ (δ^13^C) for wolf's food sources (McLaren, Crawshaw, & Patterson, 2015). For black bears, we used a TDF of 3.7 ± 0.2‰ for δ^13^C (Mowat, Curtis, & Lafferty, 2017) and the equation developed by Felicetti et al.(2003) for δ^15^N. We report the results of SIA (Simmr) as proportions of each food source in consumers' diet. To assess the effect of using prior information on the precision of diet estimates, we conducted trials with and without priors. As prior in SIA must sum to 100% of the consumer diet (Phillips et al., 2014), we used the per cent of occurrence (%PO) of prey species, that is, %FO rescaled so that the sum of all food sources was 100%. To avoid overparameterization of models with rare sources, we did not retain food sources whose %FO were below 5% for the SIA of wolves' diet (Phillips et al., 2014). We determined priors similarly for SIA of black bears using occurrence and/or %P_DM_ of detected food sources. As fishes were observed with molecular analyses but undetected via examination of undigested remains, we retained this food source within SIA of the diet of black bears, but we limited its contribution to a maximum of 1%. Each model consisted of four Markov Chain Monte Carlo of 1,000,000 iterations, tinned by 500 and with an initial discard of the first 50,000 iterations. Diagnostic assessments for SIA are available in Supplementary files S1.

### 2.6 Statistical analyses

For molecular and morphological approaches, we evaluated whether the ordinal rank attributed to each food source (based on diet estimates) aligned well between approaches using the weighted Kappa statistic (Kw) which assessed agreement between methods on an ordinal ranking scale for food sources categories (Tauler‐Ametller, Hernandez‐Matias, Pares, Pretus, & Real, 2018). Strength of agreement between methods is stated as follows: poor (Kw < 0.2), low (0.2 ≤ Kw< 0.4), moderate (0.4 ≤ Kw<0.6), and good (Kw ≥ 0.6) (Landis & Koch, 1977). For samples for which both approaches could be carried successfully, we compared the proportion of samples for which both approaches agreed by using McNemar's chi‐squared test with 2×2 contingency tables for each food source (Mumma et al., 2016). Finally, we compared the frequency of occurrence of each food source estimated by both approaches with generalized linear mixed models (lme4) with binomial distribution taking the occurrence (0, 1) of each food source within the diet of each species as the dependent variable and methods (morphological and molecular) as the independent variable. We included year of sampling as a random effect. We evaluated if the use of priors in SIA improved the precision of diet estimates based on the range of credible intervals around the mean for each food source between models with and without priors (O'Donovan et al., 2018). We retained in the results' section only SIA whose priors were included given that they reflect the optimal use of SIA (Phillips et al., 2014; Swan et al., 2020). Diet estimates for SIA without prior are available in Appendix 2. We conducted statistical analyses using R software version 3.5.1 (R Core Team, 2018).

## 3 RESULTS

### 3.1 Diet of wolf

**FIGURE 3:**
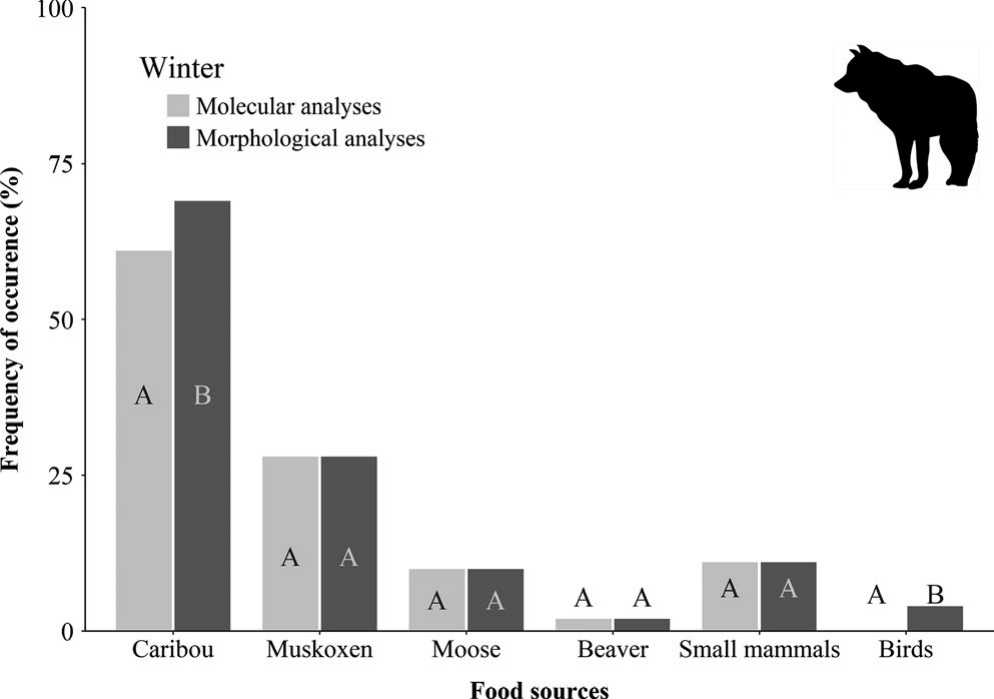
Frequency of occurrence (%FO) of food sources in the diet of wolves during winter in northern Québec (Canada) based on morphological (undigested remains) and molecular analyses (DNA barcoding) of stomach contents. Within a given food source, different letters indicate significant differences (*p* ≤.05) between approaches

Using morphological examination, we found identifiable prey remains in 79% (68 out of 86) of wolves' stomach contents, while the use of molecular tools detected preys in 83% (71 out of 86) of samples, that is, three additional stomachs for which no identifiable remain was found. Fifteen stomachs were qualified as empty using both methods and removed from the analyses. Globally, molecular and morphological methods aligned well in terms of ordinal ranking of food sources for the diet of wolves (Kw = 0.6, *p* =.001). However, we detected remains of birds in 4% of stomachs using morphological analyses, while molecular analyses failed to detect any birds' DNA. We did not find any difference in detectability between the two dietary methods for muskoxen, moose, beaver, and small mammals (Figure 3). We detected migratory caribou in a higher proportion of samples using morphological analyses compared to molecular analyses (*χ* ^2^ = 4.2 *df*=1, *p* =.04) which resulted in a higher frequency of occurrence for this food source in the diet of wolves based on morphological analyses (Figure 3).

Similar to diet estimates based on stomach content analyses, SIA showed a high reliance on ungulates during winter for wolves (Table 1). Migratory caribou dominated the diet of wolves from the Nunavik region (mean [95% CI]: 86% [81%–90%], Table 1) compared to wolves foraging in the southern part of the range that focused on moose as a primary food source (Eeyou Istchee; migratory caribou 22% [11%–33%], moose 53% [34%–77%], Table 1). Including priors in SIA enhanced the precision of diet estimates by reducing the range of credible intervals associated with most food sources (Table 1 and Appendix 2).

**TABLE 1:** Summary of stable isotope analyses for wolf diet (serum and liver, *n* = 73) for each harvest location, that is, Eeyou Istchee (*n* = 7) and Nunavik (*n* = 66), and in total

	All wolves		Eeyou Istchee		Nunavik	
Food sources^a^	$\bar{x}$	95% CI	$\bar{x}$	95% CI	$\bar{x}$	95% CI
Caribou	83	77–88	22	11–33	86	81–90
Muskoxen	9	5–14	2	0–7	11	6–15
Moose	2	0–6	53	34–77	1	0 – 2
Small mammals	6	2–10	23	8–40	2	1 – 6

Note

Results are given as proportion of food sources (mean and 95% credible intervals [CI]) in the diet of wolves at the population scale. Priors based on morphological and molecular analyses of wolves' stomach contents were included in SIA.

^a^ See Appendix 1for additional details on food sources.

### 3.2 Diet of black bear

**FIGURE 4:**
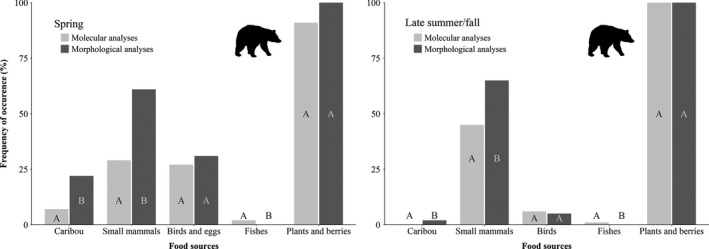
Frequency of occurrence (%FO) of food sources in the diet of black bears during spring and late summer/fall in northern Québec and Labrador (Canada) based on morphological (undigested remains) and molecular analyses (DNA barcoding) of fecal samples. Within a given food source, different letters indicate significant differences (*p* ≤ .05) between approaches

Molecular analyses could not be completed for 24% of the samples (14 out of 59) collected in spring due to sample quality, that is, DNA degradation in the samples prevented DNA of food sources to be amplified, while we could visually identify undigested remains from all of them. In comparison, 11% of samples (12 out of 105) led to no match in food sources using molecular analyses in late summer/fall. Considering this disparity between seasons, we compared occurrence of food sources and diet estimates of bears only for samples over which both methods could be completed. Although we found a moderate to good agreement using morphological and molecular analyses of feces to rank detected food sources in the diet of black bears (Spring: Kw = 0.7, *p* < .001 and Late summer/fall: Kw = 0.5, *p* = .03), we found the differences in ranking and in the detection of food sources between methods to increase at low occurrence (Figure 4). While the use of DNA barcoding allowed to detect fishes in the spring (FO = 2%) and in late summer/fall (FO = 1%) diet of black bears, it also resulted in a lower detectability and occurrence of migratory caribou (*χ* ^2^ = 7.1 *df*=1, *p* = .01) and small mammals in spring (*χ* ^2^ = 11.1 *df*=1, *p* = .001) (Figure 4). Molecular analyses also failed to detect migratory caribou DNA in feces collected in late summer/fall while morphological examination of feces revealed a frequency of occurrence of 2% for that prey (Figure 4). Small mammals were also underdetected (*χ* ^2^ = 16 *df*=1, *p* < .001) by molecular analyses for samples collected in late summer/fall compared to morphological analyses (Figure 4). Morphological analyses allowed to distinguish the contribution of calf and adult caribou (%FO, calves: 22%, adults: 5%) in the spring diet of bears. We detected birds in similar proportions of samples using both methods in spring (*χ* ^2^ = 0.5 *df*=1, *p* = .5) and late summer/fall (*χ* ^2^ = 0.5 *df*=1, *p* = .5) (Figure 4). The morphological analyses provided a distinction between eggshells and other birds remains in spring, eggshells totaling 38% of the bird remains detected. On the other hand, molecular analyses enabled finer taxonomic identification of birds compared to what was achievable with morphological analyses; the main families identified using molecular tools being *Anatidea*, *Phasianidae,* and *Fringillidae*. Although we detected plant‐based food sources in a similar proportion of samples in spring (*χ* ^2^ = 2.3 *df*=1, *p* =.13) and in all samples in late summer/fall using morphological and molecular analyses (Figure 4), the use of molecular tools allowed a finer taxonomic identification of plant items than what was achievable using the morphological method (Table 2).

**TABLE 2:** Levels of taxonomic resolution achieved for the 5 most frequently detected plant *Family* (92% of all detected DNA sequences) within the diet of black bears based on the molecular analyses of feces

Family	Genus	Species
Ericaceae	Empetrum	*Empetrum nigrum*
	Vaccinium	*Vaccinium vitis‐idea*
	Arctous	*Arctous rubra*
	Rhododendron	*Rhododendron tomentosum*
		*Rhododendron groenlandicum*
Betulaceae	Betula	–
Salicaceae	Salix	–
Cyperaceae	Carex	–
	Eriophorum	–
Poaceae	Calamagrostis	–

Note

(–) species not determined.

As observed with diet estimates based on feces, we found differences between seasonal diet using stable isotopes for black bears (Table 3). The contribution of animal‐derived proteins was higher in spring than in late summer/fall although small mammals represented more than one third of the diet during the end of the active period for bears (Table 3). Quantitative estimates of the contribution of food sources to the spring and late summer/fall diet of black bears determined by morphological analyses aligned well with seasonal diet estimates based on SIA (Table 3). Still, we detected wide variations in the contribution of some food sources to the diet of bears using both approaches (Table 3). Similar to diet estimates of wolves, including priors in SIA of the diet of black bears reduced uncertainty on the contribution of most food sources to the diet of bears (Table 3, and Appendix 2).

**TABLE 3:** Summary of seasonal stable isotope analyses (SIA) of black bears' serum (Spring: *n* = 29, Late summer/fall: *n* = 16) in northern Québec and Labrador, Canada

Food sources^a^	%P_DM_		SIA	
	$\bar{x}$	*SD*	$\bar{x}$	95% CI
Spring				
Caribou	7	18	6	0–28
Small mammals	44	41	58	28–77
Birds and eggs	4	11	5	0–23
Fishes	1	–	1	0–1
Plants and berries	44	37	30	20–42
Late summer/fall				
Caribou	1	6	1	0–8
Small mammals	39	38	36	20–49
Birds	1	8	1	0–8
Fishes	1	–	0	0–1
Plants and berries	58	38	62	49–74

Note

Results are given as mean proportion of food sources in the diet of bears at the population scale ( $\bar{x}$ ; 2016 and 2017 pooled; with 95% credible interval [CI]) for SIA. Proportions of food sources in the diet of bears derived from morphological and molecular examination of feces (%P_DM_, $\bar{x}$ with standard deviation [*SD*]) were used as priors in SIA.

^a^ See Appendix 1for additional details on food sources.

## 4 DISCUSSION

In agreement with recent reviews on dietary analyses (Horswill et al., 2018; Nielsen et al., 2018), we found that diet diversity and composition of consumers with distinct types of diet composition, for example, carnivores and omnivores, were more fully described through an approach combining the use of stable isotopes, morphological, and molecular analyses. Overall, we found that the three methods led to a consistent dietary description for wolves, an apex carnivore which has a relatively simple diet mixture, but their outcomes somewhat differed for black bears, an omnivore, which uses a wide range of potential food sources. Evaluating the outcomes of these different dietary tracing methods over a concurrent period facilitates comparisons between single‐based and multi‐technique approaches. Altogether, these findings are exportable to a variety of species and ecological contexts given samples used in this study could be collected for diet determination from a variety of taxa.

### 4.1 Joint use of morphological and molecular dietary analyses

Several studies have reported that using DNA sequencing from fecal or stomach samples increases the diversity of food sources compared to the morphological identification of undigested remains (Egeter et al., 2015; Mumma et al., 2016; Tverin et al., 2019; Xavier et al., 2018). We found that categories and ordinal ranking of food sources detected using morphological and molecular methods converged for both species. Similar results were reported for the diet composition of coyote (*Canis latrans*) and black bear by Mumma et al.(2016), although they also reported false negatives for molecular analyses. We also reported lower detection rates and frequency of occurrence of some food sources by molecular analyses into the diet of bears, and to a lesser extent for wolves. These results agree with the review of Nielsen et al.(2018) which showed that dissimilarity in diet estimates assessed by the two diet tracing methods increased when more than six potential food sources are considered.

Strict carnivores feed on fewer trophic levels compared to omnivores. Therefore, the narrow range and morphological distinctiveness of potential food sources of wolves in our northern study area could partly explain the general agreement between morphological and molecular approaches for that species. All animal prey found in the diet of wolves were easily assigned to gender or species based on hair medulla patterns. As estimates for wolves came from stomach contents, both remains and DNA detected are expected to be less degraded than what would have been uncovered in feces given the early steps of the digestive process, and absence of environmental degradation due to exposition to light or other abiotic factors (Alberdi et al., 2019; Xavier et al., 2018). Still, detection of birds' remains in stomachs of wolves was achieved with morphological but not with molecular analyses. Failure to detect that food category with molecular techniques could be linked to high heterogeneity in the distribution and quality of DNA within the stomach content (Alberdi et al., 2019). It is also worth mentioning that all stomachs in which we detected remains of birds had only few items (1–5 feathers). While those occasions resulted in an occurrence in morphological analyses, such low prevalence of remains could have depressed our ability to capture DNA fragments leading to false negatives using molecular analyses. The same explanation could be put forward for the lower detection rate of caribou by molecular analyses, a food source for which we sometimes observed only a few hairs without soft remains in the stomachs. Those issues can be expected with a species known to undertake fasting periods up to 5 days (Mech, 1970) leaving only few remains of previous meals in stomachs. Still, it is worth pointing out that quantitative metrics such as per cent biomass would be better suited than occurrence data to assess the contribution of rare food sources such as birds into the diet of wolves.

The complex diet mixture of black bears, a situation typical of omnivores, could also have led to high heterogeneity in prey DNA distribution within a given sample although we used samples taken from different locations on each feces to minimize that potential bias (Alberdi et al., 2019; Gosselin et al., 2017) and included replicates for each sample (Mata et al., 2019). Nevertheless, as pointed out for several species, molecular dietary tracing tools are well suited to highlight the presence of food items undetected by simple visual examination, for example, for items highly degraded through the digestion process (Egeter et al., 2015; Shores et al., 2015; Xavier et al., 2018). In our case, the occurrence of fishes in the diet of bears would have remained undetected without the use of molecular tools. Still, a consistent and unexpected finding in our study is that some food sources were detected in a lower proportion of feces by molecular analyses compared to the morphological approach for the diet of bears. Even though the poor distribution of DNA within samples might also apply (Alberdi et al., 2019; Mumma et al., 2016), DNA quality could also be an issue (Alberdi et al., 2019; Pompanon et al., 2012; Valentini, Pompanon, & Taberlet, 2009) and could have contributed to failures of DNA amplification or false negatives. Tollit et al.(2009) reported a lower detection rate for molecular analyses compared to visual identification of remains in feces of pinnipeds in cases involving old feces and attributed these false negatives to DNA degradation through time and/or exposition to abiotic conditions. The works of McInnes et al.(2017) on food habits of seabirds using molecular tools led to a similar finding, highlighting one important logistic limitation of using molecular analyses in free‐ranging conditions. Considering the relatively high percentage of feces samples over which molecular analyses could not be completed and that some feces were collected up to 14 days following excretion, this argument is in favor of cautiously taking into account the various effects of environmental factors on DNA degradation rates as well as sampling time for molecular dietary analyses (Alberdi et al., 2019). This also highlights that DNA degradation can happens quite fast despite samples being in a cold environment. Again, this emphasizes the need to collect feces as fresh as possible and to set correction metrics and models for false negative or imperfect detection (Alberdi et al., 2019; Monterroso et al., 2019; Morin et al., 2019).

Because time and access constraints are a common issue when dealing with free‐ranging populations, especially in remote areas, the complementary use of morphological and molecular tools during specific sampling periods or seasons allows overcoming logistic limitations. While we acknowledge temporal variation in diet according to food sources availability, our study revealed the increased detectability of diet items when using joint analyses of morphological remains and DNA sequencing. Our approach resulted in a more complete description of diet diversity for both studied species and allowed the retention of rare or undetected food items. When studying systems with few a priori information on potential and rare food sources, researchers should consider combining morphological and molecular approaches, especially for species with complex diet mixtures or with access to a wide range of potential food sources.Independently of diet composition, molecular tools could also be used in combination with morphological analyses to identify food items that could not be classified visually (e.g., soft or degraded remains) or to achieve a finer taxonomic resolution for some food categories or specific food items (Iversen et al., 2013; Lu et al., 2016; Méheust et al., 2014; Xavier et al., 2018).

### 4.2 Implementing stable isotope analyses with knowledge from independent sources

Stable isotopes are a common tool in dietary studies and have proved to be especially useful to assess animal diet over large time scales or to highlight differential foraging behavior within consumer populations (Edwards et al., 2011; Lerner et al., 2018; O'Donovan et al., 2018; Roth, 2002). Still, relying only on SIA to assess animal diet could lead to misleading results especially for species with complex diet mixtures or facing high interindividual variation in diet composition within the sampled population. Although those limitations are well documented (Caut et al., 2008; Derbridge et al., 2015; Parnell et al., 2010; Phillips et al., 2014), there are still few studies that directly compared estimates of morphological or molecular analyses of diet reconstruction with SIA over the same study period (but see Killengreen et al., 2011; Milakovic & Parker, 2011; O'Donovan et al., 2018; Resano‐Mayor et al., 2014). Reliable comparison of these methods to define the diet of animals, especially in free‐ranging conditions, is crucial in guiding the choice of an appropriate method. Nevertheless, we found good agreement in relative ranking, and contributions of food sources between SIA and other approaches for both types of diets using the results of other approaches as priors. For example, SIA and quantitative estimates based on morphological examination of remains in feces of black bears both ranked small mammals and plant‐based food sources as the main part of the diet of bears in spring and late summer/fall, respectively. A similar pattern was found from occurrence data using molecular analyses. However, for broad food categories such as plant‐based sources, the taxonomic discriminative power of SIA could not go as far as for molecular tools, and for morphological analysis to a lower extent. Still, our findings and those of others suggest that SIA are a powerful tool for dietary studies, especially those addressing the contribution of distinct food sources or food categories over long‐time scales and for longitudinal monitoring. Nevertheless, their use should be cautious depending on the studied system and research objectives because reliable estimates with SIA require a minimum of a priori information about the consumer's foraging patterns (Moore & Semmens, 2008; Swan et al., 2020).

The misuse of priors in SIA, either too broad or poorly related to the studied system, could reduce the precision and even bias diet estimates (Caut et al., 2008; Derbridge et al., 2015). For the diet of wolves and black bears, the use of empirical regional priors derived from independent sources of diet information contributed to reducing uncertainty of diet estimates obtained from SIA. This part of our works highlights that accounting for subpopulations patterns in food habits of consumers is crucial for SIA. In our study, rather than considering wolf populations as homogeneous, we achieved a more regional accurate description of their food habits by taking into account geographical subdivisions. The contribution of muskoxen in the diet of the wolf, for example, would have been overestimated by 16% in the Eeyou Istchee region and underestimated by 7% in the Nunavik region without the use of region‐specific priors in SIA (see Appendix 2). Such pattern in SIA emerged from the preliminary examination of the diet of wolves based on occurrence of food sources in stomach contents. Despite this gain in precision, we observed wide 95% CI values for certain food sources in the diet of wolves and black bears, highlighting individual patterns of food selection which are commonly observed in wildlife populations (Araujo et al., 2011; Bolnick et al., 2002). In agreement with recent studies, we therefore recommend the complementary use of approaches of diet reconstruction for studying food habits of free‐ranging animals (Horswill et al., 2018; Matley et al., 2018; Nielsen et al., 2018; O'Donovan et al., 2018).

## 5 CONCLUSION

Dietary studies aim at a wide range of objectives from defining a consumer's diet to assessing the specific contribution of a given food source in the diet of a particular species. The diversity of methodological approaches and metrics available serves well this broad range of objectives. The combined use of approaches appears as a solution to gain more accurate and unbiased insights about food habits of free‐ranging consumers independently of diet types Nielsen et al. (2018). Comparing the outcomes and limits of approaches used to study diet composition is a crucial step, and it should be repeated for various consumers living in different environments. While assessing the methods in various systems, future research should also focus on filling the gaps linked to quantitative estimation of diet composition using molecular tools (Alberdi et al., 2019; Lamb et al., 2019), as well as testing new ways of merging and scaling down dietary studies to document individual‐based diet composition and foraging patterns (Araujo et al., 2011; Musseau et al., 2020; Newsome, Garbe, Wilson, & Gehrt, 2015). The complex and structuring role of consumers from distinct trophic levels is well recognized, and a thorough understanding of their diet through multi‐approaches of diet reconstruction could be used to help understand changes in species interactions and inform wildlife management practices.

## CONFLICT OF INTEREST

None declared.

## AUTHOR CONTRIBUTIONS

**Michaël Bonin:** Conceptualization (lead); data curation (lead); formal analysis (lead); investigation (lead); methodology (lead); writing–original draft (lead); writing–review and editing (lead). **Christian Dussault:** Conceptualization (equal); formal analysis (equal); funding acquisition (equal); investigation (equal); project administration (equal); resources (equal); supervision (lead); writing–original draft (equal); writing–review and editing (equal). **Joëlle Taillon:** Conceptualization (supporting); data curation (equal); funding acquisition (equal); investigation (supporting); project administration (supporting); resources (equal); supervision (supporting); writing–original draft (equal); writing–review and editing (equal). **Nicolas Lecomte:** Formal analysis (supporting); investigation (supporting); methodology (supporting); validation (supporting); writing–original draft (equal); writing–review and editing (equal). **Steeve D. Côté:** Conceptualization (equal); funding acquisition (lead); project administration (lead); resources (lead); supervision (lead); writing–original draft (equal); writing–review and editing (equal).

## Supporting information

Supplementary Material

## Data Availability

Supporting data have been uploaded to Dryad (https://doi.org/10.5061/dryad.fqz612jq7).

## Appendix 1

### Description and isotopic profiles of food sources

**TABLE A1:** Carbon (δ^13^C) and nitrogen (δ^15^N) isotopic ratios (‰, mean ± *SD*) of food sources used in stable isotope analyses (SIA) of black bears and wolves with corresponding sample sizes (*n*) and tissue types

Food source	*n*	Tissue	δ^13^C	δ^15^N
Migratory caribou (*Rangifer tarandus*)	26	Hair	−23.5±0.4	5.1±1.2
Muskoxen (*Ovibos moschatus*)	11	Hair	−23.2±0.5	1.9±0.5
Moose (*Alces alces*)	14	Hair	−25.8±0.5	1.0±0.9
Fishes	13	Muscle	−18.2±1.5	14.7±0.9
Birds (Spring)	16	Muscle	−23.4±1.1	4.4±1.0
Birds (Late summer/fall)	5	Muscle	−23.9±0.4	2.3±0.5
Birds' eggs	3	Whole eggs	−23.7±0.5	4.4±0.5
Small mammals	8	Hair	−25.5±0.8	2.8±1.5
Plants and berries	20	Whole aerial tissue	−27.8±0.8	−3.5±2.7

Additional information:

- Sampling of food sources was carried in collaboration with the Ministère des Forêts, de la Faune et des Parcs du Québec, the Wildlife Branch of Newfoundland and Labrador Government, Cree and Inuit residents of northern Québec, and local partners. Samples were collected over the same temporal and spatial scales as samples of both consumers’ species.
- All hair and muscle samples were collected from adults.
- Isotopic ratios for *fishes*are the mean values obtained for Arctic char (*Salvelinus alpinus*) and lake trout (*Salvelinus namaycush*).
- Isotopic ratios for *birds (Spring)* are the mean values obtained from snow goose (*Anser caerulescens*), Canada goose (*Branta canadensis*), and ptarmigan (*Lagopus* spp.) and isotopic ratio for *birds (Late summer/fall)* is from ptarmigan solely.
- Isotopic ratios for *birds' eggs* are from Canada goose’ eggs.
- Isotopic ratios for *small mammals*are the mean values obtained for lemmings' spp. (*Lemmus* spp.) and arctic hare (*Lepus arcticus*).
- Isotopic ratios for *plants and berries* are the mean values obtained from *Rhododendron tomentosum, Rododendron groenladicum, Salix*spp*., Betulosa glandulosa, Vaccinium vittis‐idea, Vaccinium uliginosum, Empetrum nigrum, Empetrum rubrum, Equisetum*spp*., Arctous alpina, Arctous rubra, Carex*spp*., Poa arctica,*and*Calamagrostis*spp. We selected these plant species based on field observations, and results of morphological and molecular analyses on feces for black bears.

## Appendix 2

### SIA for wolves and bears without prior.

**TABLE B1:** Summary of stable isotope analyses for wolf diet (serum and liver, *n* = 73) for each harvest location, that is, Eeyou Istchee (*n* = 7) and Nunavik (*n* = 66), and in total

Food sources^a^, ^a^	All wolves		Eeyou Istchee		Nunavik	
	$\bar{x}$	95% CI	$\bar{x}$	95% CI	$\bar{x}$	95% CI
Caribou	88	82–93	12	3–24	92	87–96
Muskoxen	3	1–8	18	3–35	4	1–9
Moose	3	1–8	50	3–46	2	0–5
Small mammals	6	1–10	20	27–68	2	1–5

Note

Results are given as proportion of food sources (mean and 95% credible intervals [CI]) for models without priors.

^a^ See Appendix 1 for additional details on food sources.

**TABLE B2:** Summary of seasonal stable isotope analyses (SIA) of black bears' serum (Spring: *n* = 29, Late summer/fall: *n* = 16) in northern Québec and Labrador, Canada

Food sources^a^, ^a^	Spring		Late summer/fall	
	$\bar{x}$	95% CI	$\bar{x}$	95% CI
Caribou	9	1–28	6	1–16
Small mammals	39	8–65	16	2–35
Birds and/or eggs	8	1–21	6	1–16
Fishes	4	0–10	2	0–7
Plants and berries	40	25–56	70	56–82

Note

Results are given as mean proportion of food sources in the diet of bears ( $\bar{x}$ ; 2016 and 2017 pooled; with 95% credible interval [CI]) for models without priors.

^a^ See Appendix 1 for additional details on food sources.
