# Supplementary material for: Combining stable isotopes, morphological, and molecular analyses to reconstruct the diet of free‐ranging consumers
Source: Ecol Evol. 2020 May 27;10(13):6664–76. doi: 10.1002/ece3.6397 (PMC7381590; doi:10.1002/ece3.6397)
Supplement: Supplementary file 1 — Supplementary Material [file ECE3-10-6664-s001.docx]

**Supporting Information for: Combining stable isotopes, morphological, and molecular analyses to reconstruct the diet of free-ranging consumers.**

Michaël Bonin^1*^, Christian Dussault^1,2^, Joëlle Taillon^1,2^, Nicolas Lecomte^1,3^ and Steeve D. Côté^1^

1. Caribou Ungava, département de biologie, Centre d’études nordiques, Université Laval, 1045 avenue de la Médecine, Québec, QC G1V 0A6, Canada
2. Direction de l’expertise sur la faune terrestre, l’herpétofaune et l’avifaune, Ministère des Forêts, de la Faune et des Parcs, 880 chemin Sainte-Foy, Québec, QC G1S 4X4, Canada
3. Chaire de recherche du Canada en écologie polaire et boréale, département de biologie, Université de Moncton, 18 avenue Antonine-Maillet, Moncton NB E1A 3E9, Canada

***Corresponding author and present address:**

Michaël Bonin

1045 avenue de la Médecine, Pavillon Alexandre-Vachon room 3076

Université Laval, QC, G1V 0A6, Canada

Phone: (418) 656-2131 ext. 408152

Email: michael.bonin.1@ulaval.ca

**Supporting Information S1. Additional details on diagnostic and assessment for stable isotope models.**

All isotopic analyses were realized using the Simmr package (Andrew Parnell. 2019. Simmr: A stable isotope mixing model. R package version 0.4.1. https://CRAN.R-Project.org/package=simmr) using R software version 3.5.1 (R Core Team 2018).

*Black bear: Diagnostic assessment for SIA*

Table S1.1. Gelman diagnostic for isotopic analyses of serum samples for black bear addressing diet composition of bears during spring and late summer/fall with and without prior.

| Food sources  and tracers | Gelman values^1^ | | | |
| --- | --- | --- | --- | --- |
|  | No prior - Spring | With prior -  Spring | No prior - Late summer/fall | With prior - Late summer/fall |
| Caribou | 1 | 1 | 1 | 1 |
| Fishes | 1 | 1 | 1 | 1 |
| Small mammals | 1 | 1 | 1 | 1 |
| Birds and/or eggs | 1 | 1 | 1 | 1 |
| Plants and berries | 1 | 1 | 1 | 1 |
| δ^13^C | 1 | 1 | 1 | 1 |
| δ^15^N | 1 | 1.01 | 1 | 1 |

^1^ Gelman values: Values should all be close to 1.


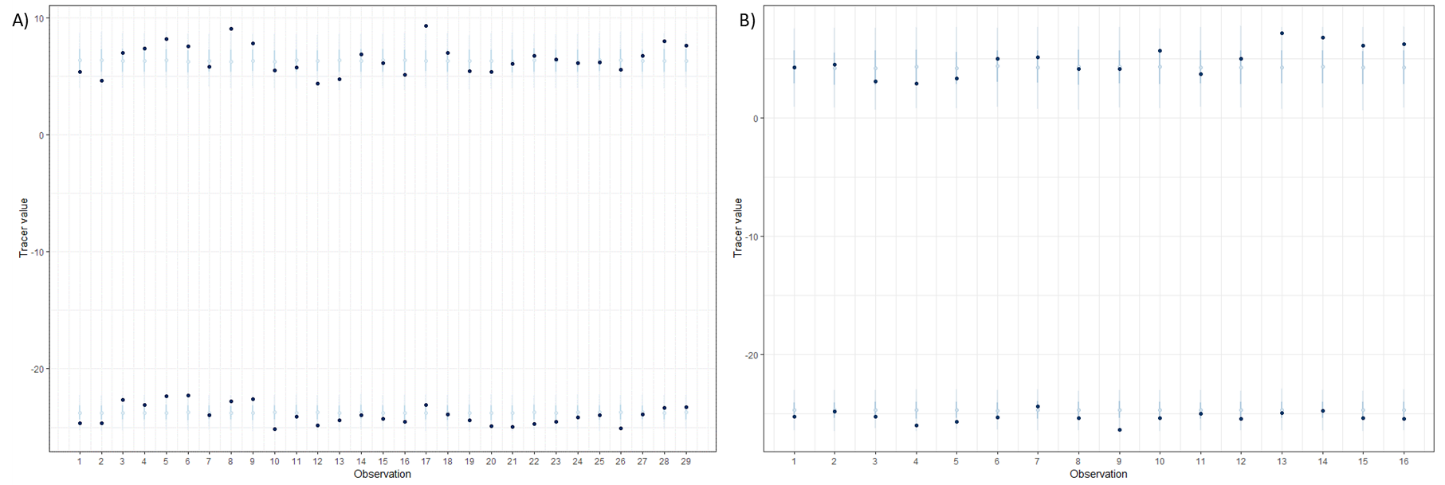


Figure S1.1. Posterior predictive distribution (probability interval for posterior predictors set to 0.5) allowing visualisation of model fit (solid dots: data points, shaded dots: fitted values) for isotopic analyses of serum samples for black bear addressing diet composition of bears during: A) spring, and B) late summer/fall.

Table S1.2. Correlation matrix for food sources’ contribution in isotopic analyses of serum samples for black bear addressing diet composition of bears during spring without prior.

|  | Caribou | Fishes | Small mammals | Plants and berries | Birds and eggs | δ^13^C | δ^15^N |
| --- | --- | --- | --- | --- | --- | --- | --- |
| Caribou | 1.00 | -0.13 | -0.64 | 0.41 | -0.09 | 0.20 | -0.02 |
| Fishes | -0.13 | 1.00 | -0.40 | 0.62 | -0.11 | 0.09 | -0.06 |
| Small mammals | -0.64 | -0.40 | 1.00 | -0.90 | -0.41 | -0.28 | 0.01 |
| Plants and berries | 0.41 | 0.62 | -0.90 | 1.00 | 0.20 | 0.16 | -0.03 |
| Birds and eggs | -0.09 | -0.11 | -0.41 | 0.20 | 1.00 | 0.22 | 0.05 |
| δ^13^C | 0.20 | 0.09 | -0.28 | 0.16 | 0.22 | 1.00 | -0.01 |
| δ^15^N | -0.02 | -0.06 | 0.01 | -0.03 | 0.05 | -0.01 | 1.00 |

Table S1.3. Correlation matrix of food sources’ contribution in isotopic analyses of serum samples for black bear addressing diet composition of bears during spring with prior.

|  | Caribou | Fishes | Small mammals | Plants and berries | Birds and eggs | δ^13^C | δ^15^N |
| --- | --- | --- | --- | --- | --- | --- | --- |
| Caribou | 1.00 | -0.47 | -0.74 | 0.58 | -0.34 | 0.19 | -0.02 |
| Fishes | -0.47 | 1.00 | 0.59 | -0.49 | -0.19 | -0.16 | -0.02 |
| Small mammals | -0.74 | 0.59 | 1.00 | -0.88 | -0.30 | -0.25 | -0.06 |
| Plants and berries | 0.58 | -0.49 | -0.88 | 1.00 | 0.12 | 0.19 | 0.04 |
| Birds and eggs | -0.34 | -0.19 | -0.30 | 0.12 | 1.00 | 0.11 | 0.12 |
| δ^13^C | 0.19 | -0.16 | -0.25 | 0.19 | 0.11 | 1.00 | 0 |
| δ^15^N | -0.02 | -0.02 | -0.06 | 0.04 | 0.12 | 0 | 1.00 |

Table S1.4. Correlation matrix of food sources’ contribution in isotopic analyses of serum samples for black bear addressing diet composition of bears during late summer/fall without prior.

|  | Caribou | Fishes | Small mammals | Plants and berries | Birds | δ^13^C | δ^15^N |
| --- | --- | --- | --- | --- | --- | --- | --- |
| Caribou | 1.00 | -0.14 | -0.39 | 0.05 | -0.12 | 0.09 | -0.02 |
| Fishes | -0.14 | 1.00 | -0.35 | 0.34 | -0.08 | 0.12 | -0.01 |
| Small mammals | -0.39 | -0.35 | 1.00 | -0.80 | -0.36 | 0.02 | -0.07 |
| Plants and berries | 0.05 | 0.34 | -0.80 | 1.00 | -0.05 | -0.20 | 0.12 |
| Birds | -0.12 | -0.08 | -0.36 | -0.05 | 1.00 | 0.17 | -0.02 |
| δ^13^C | 0.09 | 0.12 | 0.02 | -0.20 | 0.17 | 1.00 | -0.07 |
| δ^15^N | -0.02 | -0.01 | -0.07 | 0.12 | -0.02 | -0.07 | 1.00 |

Table S1.5. Correlation matrix of food sources’ contribution in isotopic analyses of serum samples for black bear addressing diet composition of bears during late summer/fall with prior.

|  | Caribou | Fishes | Small mammals | Plants and berries | Birds | δ^13^C | δ^15^N |
| --- | --- | --- | --- | --- | --- | --- | --- |
| Caribou | 1.00 | 0.15 | -0.42 | 0.18 | -0.06 | 0.03 | -0.02 |
| Fishes | 0.15 | 1.00 | -0.75 | 0.82 | 0.05 | -0.13 | 0.09 |
| Small mammals | -0.42 | -0.75 | 1.00 | -0.92 | -0.36 | -0.11 | -0.10 |
| Plants and berries | 0.18 | 0.82 | 0.92 | 1.00 | 0.08 | -0.15 | 0.11 |
| Birds | -0.06 | 0.05 | -0.36 | 0.08 | 1.00 | 0.04 | 0.06 |
| δ^13^C | 0.03 | -0.13 | 0.11 | -0.15 | 0.04 | 1.00 | -0.01 |
| δ^15^N | -0.02 | 0.09 | -0.10 | 0.11 | 0.06 | -0.01 | 1.00 |

*Wolves: Diagnostic assessment for SIA*

Table S1.6. Gelman diagnostic for isotopic analyses addressing diet composition of wolves (all individuals, Eeyou Istchee, and Nunavik) with and without prior.

| Wolves SIA | Gelman values^1^ | | | | | |
| --- | --- | --- | --- | --- | --- | --- |
|  | Caribou | Muskoxen | Small mammals | Moose | δ^13^C | δ^15^N |
| All - without prior | 1 | 1 | 1 | 1 | 1 | 1 |
| All - with prior | 1 | 1 | 1 | 1 | 1 | 1 |
| Eeyou Istchee - without prior | 1 | 1 | 1 | 1 | 1 | 1 |
| Eeyou Istchee - with prior | 1 | 1 | 1 | 1 | 1 | 1 |
| Nunavik - withour prior | 1 | 1 | 1 | 1 | 1 | 1 |
| Nunavik - with prior | 1 | 1 | 1 | 1 | 1 | 1 |

^1^ Gelman values: Values should all be close to 1.


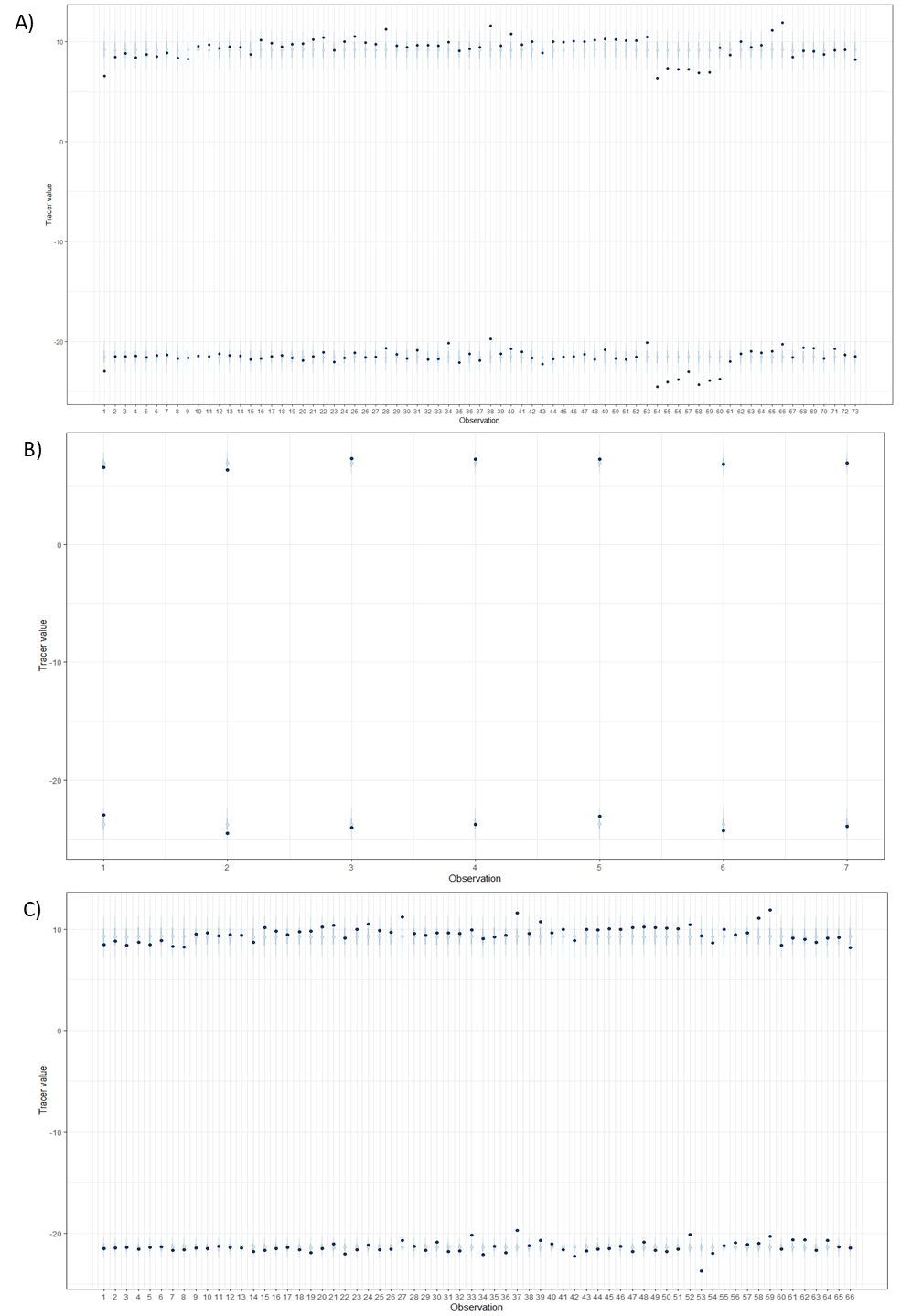


Figure S1.2. Posterior predictive distribution (probability interval for posterior predictors set to 0.5) allowing visualisation of model fit (solid dots: data points, shaded dots: fitted values) for isotopic analyses addressing diet composition of wolves: A) all individuals, B) wolves from Eeyou Istchee, and C) wolves from Nunavik.

Table S1.7. Correlation matrix of food sources’ contribution in isotopic analyses addressing diet composition of wolves (all individuals) without prior.

|  | Caribou | Muskoxen | Small mammals | Moose | δ^13^C | δ^15^N |
| --- | --- | --- | --- | --- | --- | --- |
| Caribou | 1.00 | -0.56 | -0.49 | -0.27 | 0.0 | -0.24 |
| Muskoxen | -0.56 | 1.00 | -0.07 | -0.14 | 0.1 | 0.15 |
| Small mammals | -0.49 | -0.07 | 1.00 | -0.42 | 0.0 | 0.08 |
| Moose | -0.27 | -0.14 | -0.42 | 1.00 | 0.0 | 0.10 |
| δ^13^C | -0.04 | 0.06 | -0.02 | 0.02 | 1.0 | 0.02 |
| δ^15^N | -0.24 | 0.15 | 0.08 | 0.10 | 0.0 | 1.00 |

Table S1.8. Correlation matrix of food sources’ contribution in isotopic analyses addressing diet composition of wolves (all individuals) with prior.

|  | Caribou | Muskoxen | Small mammals | Moose | δ^13^C | δ^15^N |
| --- | --- | --- | --- | --- | --- | --- |
| Caribou | 1.00 | -0.66 | -0.49 | -0.1 | -0.04 | -0.40 |
| Muskoxen | -0.66 | 1.00 | -0.17 | -0.17 | 0.08 | 0.28 |
| Small mammals | -0.49 | -0.17 | 1.00 | -0.31 | -0.06 | 0.15 |
| Moose | -0.10 | -0.17 | -0.31 | 1.00 | 0.03 | 0.08 |
| δ^13^C | -0.04 | 0.08 | -0.06 | 0.03 | 1.0 | 0.04 |
| δ^15^N | -0.40 | 0.28 | 0.15 | 0.08 | 0.04 | 1.00 |

Table S1.9. Correlation matrix of food sources’ contribution in isotopic analyses addressing diet composition of wolves (Eeyou Istchee) without prior.

|  | Caribou | Muskoxen | Small mammals | Moose | δ^13^C | δ^15^N |
| --- | --- | --- | --- | --- | --- | --- |
| Caribou | 1.00 | -0.54 | -0.49 | 0.37 | 0.11 | 0.04 |
| Muskoxen | -0.54 | 1.00 | 0.17 | -0.65 | 0.09 | -0.05 |
| Small mammals | -0.49 | 0.17 | 1.00 | -0.78 | -0.20 | -0.04 |
| Moose | 0.37 | -0.65 | -0.78 | 1.00 | 0.06 | 0.05 |
| δ^13^C | 0.11 | 0.09 | -0.20 | 0.06 | 1.00 | 0 |
| δ^15^N | 0.04 | -0.05 | -0.04 | 0.05 | 0 | 1.00 |

Table S1.10. Correlation matrix of food sources’ contribution in isotopic analyses addressing diet composition of wolves (Eeyou Istchee) with prior.

|  | Caribou | Muskoxen | Small mammals | Moose | δ^13^C | δ^15^N |
| --- | --- | --- | --- | --- | --- | --- |
| Caribou | 1.00 | -0.19 | -0.37 | -0.07 | 0.21 | -0.09 |
| Muskoxen | -0.19 | 1.00 | 0.06 | -0.26 | 0.10 | -0.04 |
| Small mammals | -0.37 | 0.06 | 1.00 | -0.86 | -0.18 | -0.11 |
| Moose | -0.07 | -0.26 | -0.86 | 1.00 | 0.05 | 0.16 |
| δ^13^C | 0.21 | 0.10 | -0.18 | 0.05 | 1.00 | -0.03 |
| δ^15^N | -0.09 | -0.04 | -0.11 | 0.16 | -0.03 | 1.00 |

Table S1.11. Correlation matrix of food sources’ contribution in isotopic analyses addressing diet composition of wolves (Nunavik) without prior.

|  | Caribou | Muskoxen | Small mammals | Moose | δ^13^C | δ^15^N |
| --- | --- | --- | --- | --- | --- | --- |
| Caribou | 1.00 | -0.80 | -0.37 | -0.31 | -0.23 | -0.07 |
| Muskoxen | -0.80 | 1.00 | 0.03 | -0.11 | 0.16 | 0.04 |
| Small mammals | -0.37 | 0.03 | 1.00 | -0.24 | 0.12 | 0.02 |
| Moose | -0.31 | -0.11 | -0.24 | 1.00 | 0.08 | 0.07 |
| δ^13^C | -0.23 | 0.16 | 0.12 | 0.08 | 1.00 | 0 |
| δ^15^N | -0.07 | 0.04 | 0.02 | 0.07 | 0 | 1.00 |

Table S1.12. Correlation matrix of food sources’ contribution in isotopic analyses addressing diet composition of wolves (Nunavik) with prior.

|  | Caribou | Muskoxen | Small mammals | Moose | δ^13^C | δ^15^N |
| --- | --- | --- | --- | --- | --- | --- |
| Caribou | 1.00 | -0.86 | -0.40 | -0.06 | -0.22 | -0.10 |
| Muskoxen | -0.86 | 1.00 | -0.08 | -0.09 | 0.18 | 0.08 |
| Small mammals | -0.40 | -0.08 | 1.00 | -0.15 | 0.10 | 0.04 |
| Moose | -0.06 | -0.09 | -0.15 | 1.00 | 0.01 | 0.04 |
| δ^13^C | -0.22 | 0.18 | 0.10 | 0.01 | 1.00 | 0.04 |
| δ^15^N | -0.10 | 0.08 | 0.04 | 0.04 | 0.04 | 1.00 |
